# Local IGFBP-3 mRNA expression, apoptosis and risk of colorectal adenomas

**DOI:** 10.1186/1471-2407-8-143

**Published:** 2008-05-22

**Authors:** Temitope O Keku, Robert S Sandler, James G Simmons, Joseph Galanko, John T Woosley, Michelle Proffitt, Oluwaseun Omofoye, Maya McDoom, Pauline K Lund

**Affiliations:** 1 Department of Medicine and Center for Gastrointestinal Biology & Disease, School of Medicine, University of North Carolina, Chapel Hill, North Carolina, USA; 2 Department of Cell and Molecular Physiology, School of Medicine, University of North Carolina, Chapel Hill North Carolina, USA; 3 Department of Surgical Pathology, School of Medicine, University of North Carolina, Chapel Hill, North Carolina, USA

## Abstract

**Background:**

IGF binding protein-3 (IGFBP-3) regulates the bioavailability of insulin-like growth factors I and II, and has both anti-proliferative and pro-apoptotic properties. Elevated plasma IGFBP-3 has been associated with reduced risk of colorectal cancer (CRC), but the role of tissue IGFBP-3 is not well defined. We evaluated the association between tissue or plasma IGFBP-3 and risk of colorectal adenomas or low apoptosis.

**Methods:**

Subjects were consenting patients who underwent a clinically indicated colonoscopy at UNC Hospitals and provided information on diet and lifestyle. IGFBP-3 mRNA in normal colon was assessed by real time RT-PCR. Plasma IGFBP-3 was measured by ELISA and apoptosis was determined by morphology on H & E slides. Logistic regression was used to compute odds ratio (OR) and 95% confidence intervals.

**Results:**

We observed a modest correlation between plasma IGFBP-3 and tissue IGFBP-3 expression (p = 0.007). There was no significant association between plasma IGFBP-3 and adenomas or apoptosis. Tissue IGFBP-3 mRNA expression was significantly lower in cases than controls. Subjects in the lowest three quartiles of tissue IGFBP-3 gene expression were more likely to have adenomas. Consistent with previous reports, low apoptosis was significantly associated with increased risk of adenomas (p = 0.003). Surprisingly, local IGFBP-3 mRNA expression was inversely associated with apoptosis.

**Conclusion:**

Low expression of IGFBP-3 mRNA in normal colonic mucosa predicts increased risk of adenomas. Our findings suggest that local IGFBP-3 in the colon may directly increase adenoma risk but IGFBP-3 may act through a pathway other than apoptosis to influence adenoma risk.

## Background

The insulin-like growth factors, IGF-I and IGF-II, and their corresponding receptors play important roles in proliferation, apoptosis and differentiation in normal and malignant cells. The IGFs exert their growth promoting effects through the type 1 IGF-receptors [1] A family of at least six insulin-like growth factor binding proteins (IGFBPs) exist in the circulation and tissues and bind to the IGFs with high affinity [2]. A major role of these IGFBPs is to regulate the bioavailability of IGFs for interaction with the type 1 IGF receptor [2]. IGFBP-3 is the predominant IGF binding protein in plasma and together with the acid labile subunit (ALS) sequesters approximately 90% of the IGFs in to a 150 kDA complex that does not cross capillary membranes. IGF complexes also exist as approximately 50 kDA complexes comprising IGFs and IGFBPs that can exit capillaries. IGFBP-3 exists as a 43–45 kDa isoform with high affinity for the IGFs or as an inactive 30 kDa proteolytic cleaved fragment [1,3,4].

IGFBP-3 regulates cell growth by IGF-dependent [5] and IGF-independent mechanisms [6-10]. IGFBP-3 induces apoptosis and inhibits proliferation in human breast, lung, prostate and colon cancer cells in vitro [6-10] and in experimental animal models of colon carcinoma [11]. IGFBP-3 is induced by p53 in colon cancer cell lines and is thought to play a role in anti-proliferative or pro-apoptotic actions of p53 [12]. In some systems, IGFBP-3 is induced by TGF-β and plays a role in TGF-β induced apoptosis [5,6,13-15]. The mechanisms for the IGF-independent actions of IGFBP-3 on cell functions are not fully understood but may relate to the nuclear actions of IGFBP-3. IGFBP-3 can translocate to the nucleus [16-18] to regulate cell growth and modulate the expression of genes associated with proliferation and apoptosis [7,19].

Epidemiological studies support an association between elevated circulating levels of IGF-I and reduced IGFBP-3 levels in the circulation and increased risk of breast [20,21], prostate [22], and colorectal cancer or adenoma [23-26]. However, this is not consistent in all studies [27-30]). In addition to regulating the bioavailability of plasma IGFs, IGFBP3 is expressed locally in most if not all tissues including the intestine [31]. The contribution of locally expressed IGFBP-3 to pre-malignant and malignant lesions in the colon is not well understood and few studies have evaluated tissue expression of IGFBP-3 in relation to cancer development and progression. It is also not clear whether plasma levels of IGFBP-3 reflect levels of expression in particular tissues such as the colon.

The present study builds on previously published findings that low apoptosis in normal mucosa predicts elevated risk of colorectal adenomas [32]. In the study reported here, we evaluated the associations of plasma IGFBP-3, and local IGFBP-3 mRNA expression with colorectal adenomas or apoptosis in normal colonic mucosa. We tested the hypothesis that low levels of plasma or tissue IGFBP-3 will predict increased risk of adenomas and low apoptosis in normal colonic mucosa.

## Methods

### Study Population

The study population included consenting patients enrolled in the Diet and Health Study (DHS) IV, a hospital-based cross sectional study of patients who underwent colonoscopy for a variety of indications (39%) or screening (61%) between November 2001 and December 2002 at the University of North Carolina Hospitals (UNCH). Participants were diverse with respect to race, socioeconomic status and religion. Between November 2001 and December 2002, a total of 3161 outpatient colonoscopies were performed at UNC Hospitals of which 1925 subjects were ineligible. Reasons for exclusion in the study were incomplete examination (cecum not reached), age < 30 years, inability to give informed consent, polyposis (>100 polyps), previous colon resection or cancer, colitis (such as ulcerative colitis and Crohn's disease) and previous colon adenoma. Eligible participants provided informed consent, agreed to a telephone interview, gave rectal biopsies during the procedure and had blood drawn. The study was approved by the Institutional Review Board (IRB) at the University of North Carolina School of Medicine (Protocol #05-3138).

There were 1236 subjects eligible for the study, among whom 132 refused and 274 were not asked to participate because the research assistant was not available. A total of 830 eligible subjects agreed to participate in the study of which 706 completed at least one questionnaire, 669 gave blood samples or biopsies or both. 580 had adequate RNA quantity and quality for IGFBP-3 gene expression analysis, and 461 had apoptosis data. The study pathologist assessed and classified all colon polyps in the study using standard pathological criteria. Cases were defined as individuals who had one or more adenomatous polyps (n = 164) and control subjects had no adenomatous polyps (n = 416). There were no significant differences in demographic characteristics between eligible subjects who participated in the study and those that did not.

### Data Collection

Eligible colonoscopy patients were approached between 7:30 AM and 2:30 PM. This time was chosen to allow adequate time for transporting and processing blood specimens in the laboratory. At the time of colonoscopy, the research assistant obtained consent from eligible participants, asked about the time they had their last meal and the type of colonoscopy prep used. The assistant also measured their waist, hips, height and weight. Information about lifestyle/diet, demographics, family history, education, medical history, physical activity, and other environmental factors were collected using a pre-coded questionnaire during subsequent telephone interview. Telephone interview was scheduled usually at a time convenient for the subject but no later than 12 weeks after the colonoscopy. Diet was assessed with the NCI quantitative Diet History Questionnaire version 1.0 (NIH Applied Research Program, National Cancer Institute 2002). Participants were asked to recall foods eaten, the frequency at which they were eaten, and serving size. The nutrient information was obtained from the NCI nutrient analysis database and program (NIH Applied Research Program, National Cancer Institute 2002; [33]). Lifestyle and dietary data were available from 678 subjects.

### Biological Specimens and Laboratory Assays

The biopsy collection procedure was similar to previously described methods [32,34]. Briefly, subjects used either a balanced electrolyte polyethylene glycol lavage or a phosphate-containing purge for colonoscopy preparation. At the beginning of the colonoscopy procedure, standard disposable, fenetrated colonoscopy forceps (Wilson-Cook, Winston-Salem, NC) were used to obtain six mucosal biopsies 8–10 cm from the anal verge. Biopsies were taken one-at-a-time from normal appearing mucosa taking care to avoid raised lesions or larger blood vessels. Sample sites were as uniform as possible across patients. Blood samples were obtained from participants at the time of colonoscopy prior to placing an IV line, and usually between 7:30 AM and 2:30 PM. Before transportation to the laboratory, blood specimens were kept at 4°C and were processed immediately on arrival. Plasma was separated from the blood samples and stored in multiple aliquots at -80°C until assayed. Care was taken to avoid repeated freezing and thawing of samples. Based on verbal response about last food intake and supportive evidence of a clean colon, only samples from patients with excellent colonoscopy preparation and confirmed overnight fast were analyzed.

### RNA Extraction

Four colon biopsy specimens from each participating subject were flash frozen at and stored at -80°C until extracted. Total RNA was extracted from colon tissue specimens using RNeasy kit (Qiagen, Valencia, CA) following the manufacturer's protocol. Briefly, the tissue was homogenized at 12,000 rpm in guanidine isothiocyanate (GITC) containing buffer (Qiagen, Valencia, CA) using Polytron PT-MR-3100 homogenizer with disposable homogenizer probes to prevent cross sample contamination. The homogenate was centrifuged at 10,000 rpm for 3 minutes. The supernatant was collected and 75% ethanol was added to the sample to provide appropriate binding conditions and then applied to an RNeasy spin column followed by centrifugation at 8000 rpm for 15 seconds. The column was washed three times with buffer and the RNA was eluted in RNAse free sterile water. Total RNA concentrations were quantified using a spectrophotometer at 260 nm. Mean RNA yield was 25.9 μg and was 1.1–62.0 μg. The integrity of the RNA was verified by electrophoresis on ethidium bromide/agarose gels. Only samples with intact 28S and 18S RNA and no evidence of degradation were used in this study. RNA samples were stored in aliquots at -80°C until used for reverse transcription and PCR.

### Reverse Transcription (RT) and Real Time PCR

Reverse transcription was performed according to manufacturer's instructions (AMV Reverse Transcriptase, Promega Corporation, Madison, WI). Specifically, 1 μg of total RNA from each human colon biopsy was reverse transcribed in a reaction mixture that contained 0.5 μg Oligo (dT) primer in nuclease-free water (total volume of 11.5 μl), 15 units AMV reverse transcriptase (Promega,) 4 μl of 5× transcription buffer, 2 μl of 10 mM dNTPs, and 1 μl RNasin. The 20 μl reactions were incubated at 42°C for 1 hr followed by addition of 30 μl of nuclease-free water and then heat-inactivated at 90°C for 3 minutes. A sample of each total RNA was also processed through identical reverse transcription reactions but without addition of reverse transcriptase. This reaction was used as a control to ensure that samples were free of contaminating DNA. None of the samples showed evidence of DNA contamination. The cDNA concentrations were determined by spectrophotometry and aliquots were stored at -80°C until used for PCR.

IGFBP-3 abundance was determined by LightCycler-based (Roche Applied Science, Indianapolis IN) real time polymerase chain reaction (PCR). Specific IGFBP-3 forward and reverse primers and two fluorogenic hybridization probes, one labeled with fluorescein and the other with Light-Cycler Red 640 were designed using the LightCycler Primer-Probe design software 2.0. IGFBP-3 primers and probes were purchased from Idaho Technologies (Idaho Technologies, Idaho Falls ID). The sequences used to amplify a 195-bp fragment containing spanning exons 2 and exon 3 of IGFBP-3 were as follows: forward 5' AAATGCTAGTGAGTCGG 3'; reverse 5'TGTCTATGGGTCTTGAA 3'; probe 1, 5' GATAATCATCATCAAGAAAGGGCA-fluorescein-3'; probe 2, 5'-LC-640 red-CTAAAGACAGCCAGCGC 3'. The PCR reaction mix consisted of PCR grade water to 20 μl, MgCl_2 _(4 mM), forward primer (0.5 μM), reverse primer (0.5 μM), LightCycler Fast Start DNA Master Hybprobe mix (1×), probe 1 (0.2 μM) and probe 2 (0.4 μM) and 2 μl of cDNA template. Amplification in the LightCycler included an initial denaturation at 95°C for 10 minutes followed by 45 cycles of denaturation at 95°C for 10 seconds, annealing of forward and reverse primers to target cDNA at 57°C for 10 seconds, and extension at 72°C for 5 seconds. During annealing, the fluorescent probes hybridize with the product bringing the fluorescein and RED 640 probes into proximity. The two fluorescent tags communicate through FRET (fluorescent resonance transfer) leading to Red 640 emission, which is directly proportional to the amount of target at that amplification cycle.

Standards, positive and negative controls (water and non-reverse transcribed sample) were included with PCR amplification runs. Three internal controls were included in each assay run. All reactions were run in triplicate on the LightCycler 32 capillary equipment. Porphobilingen deaminase (PBGD), a low abundance housekeeping gene that is expressed at constant levels in a wide range of tissues was used as reference. The primers and probes for porphobilingen deaminase were purchased commercially (Roche LightCycler hPBGD housekeeping gene set) and the sequences are not available for proprietary reasons (Roche Applied Science). Relative quantification was determined using the LightCycler Relative Quantification software (Roche Applied Science). An external cDNA standard (calibrator) prepared from pooled RNA from control subjects was used for relative quantification in each PCR run. This pooled RNA also served as a constant calibration point between PCR runs. The normalized ratio for IGFBP3 abundance was calculated as the ratio of IGFBP-3/PBGD for each sample divided by the IGFBP-3/PBGD ratio of the calibrator. The intra and inter coefficient of variation for internal standards used in every assay run were 0.7 and 3.3% for IGFBP-3 and 1.4 and 6.8% for PBGD. No PCR products were detected when the reverse transcriptase step was omitted.

### Enzyme Immunoassays

Plasma IGFBP-3 was measured by enzyme-linked immunosorbent (ELISA) assay using reagents from Diagnostic Systems Laboratory, (Webster, Texas) according to the manufacturer's instructions as previously described [34]. Samples were run in duplicate and the laboratory personnel were blinded to the case or control status of samples. Intra-assay and inter-assay coefficients of variation for IGFBP-3 were 4.7% and 10.2% respectively.

### Assays of Apoptosis

Briefly, two colonic biopsies were fixed in 10% buffered formalin and processed by routine histology. We assessed apoptosis by morphological identification of apoptotic cells on H & E stained sections in biopsies from normal colonic mucosa. Apoptosis was confirmed on a subset of samples by the Terminal deoxynucleotidyl Transferase Biotin-dUTP Nick End Labeling (TUNEL) assay (ApoTag, Intergen, Purchase, NY) as described previously [32,34]. Longitudinal crypt sections, 8–12 per biopsy were selected and scored using previously described criteria [32,34,35]. Apoptosis was observed in isolated single epithelial cells, distinct from lamina propria immune cells on H & E stained sections. Apoptotic cells were recognized by cell shrinkage, chromatin condensation and formation of apoptotic bodies. Cells were not scored as apoptotic if the nucleus did not meet these criteria. Apoptosis was scored in the tissue sections by an experienced technician who was blinded to the case-control status of the subjects. The same reader scored selected masked slides a second time. The intra-reader variability for morphologic determination of apoptosis was less than 1%. Apoptosis was expressed as the average number of apoptotic bodies per crypt counting at least 16 (and as many as 24) crypts from 2 biopsies per subject.

### Statistical Analysis

For continuous variables, means and standard errors were computed and cases and controls were compared using a t-test. Comparison of continuous and categorical variables between adenoma cases and non-adenoma controls were made using t-tests and Fishers Exact Test respectively. Mean plasma IGFBP-3 or tissue IGFBP-3 mRNA was compared between case and control subjects using t-tests. Correlation of plasma IGFBP-3 and tissue IGFBP-3 was assessed by Spearman's correlation coefficient. The distribution of IGFBP-3 measures among control subjects were used to generate quartile values. The highest quartile of plasma or tissue IGFBP-3 was considered as the reference. Apoptosis was expressed as the average number of apoptotic bodies per crypt as described above. Using the median in controls as cut point, we divided apoptosis measures into low (below the median; quartiles 1 and 2 combined) and high (above the median; quartiles 3 and 4 combined) categories.

Potential confounders considered in the models were race (white/black), sex, smoking status (current/former/never), family history of colorectal cancer (yes/no), age, waist/hip ratio, body mass index (BMI), NSAID usage (uses per month over the previous five years), physical activity (MET-minutes per day), daily calories, daily alcohol intake (grams per day) daily calcium (grams per day), red meat intake (ounces per day) and fiber (grams per day). Each co-variable was added to a logistic regression model with adenoma case status as the response and the variable of interest (plasma IGFBP-3 or tissue IGFBP-3) as a predictor. A covariable was considered a potential confounder if it changed the parameter estimate by at least 10%. All such variables were entered into a model and a backwards stepwise procedure was performed to determine the final model. Similar analyses were used to examine the association between apoptosis and adenomas. Apoptosis was divided into two groups (lower half and upper half) and was the predictor of interest. Similar procedures were used for analysis of associations between apoptosis and plasma or tissue IGFBP-3, except apoptosis was the response instead of adenoma status and IGFBP-3 variable divided into quartiles was the predictor.

## Results

**Figure 1:**
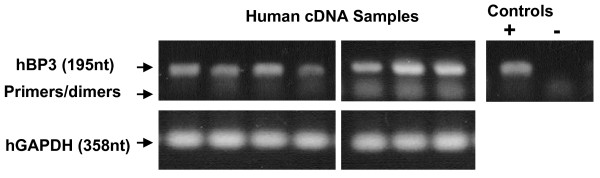
**RT-PCR demonstrates human 195-nucleotide (nt) IGFBP-3 products using cDNA transcribed from human colonic biopsy RNA.** cDNA samples were prepared as described in Methods. GAPDH control (358 nt) is shown below for human samples. Controls at left are PCR products using IGFBP-3 standard (+ Control) and 'no reverse transcriptase (RT)' (-Control) cDNAs.

**Figure 2:**
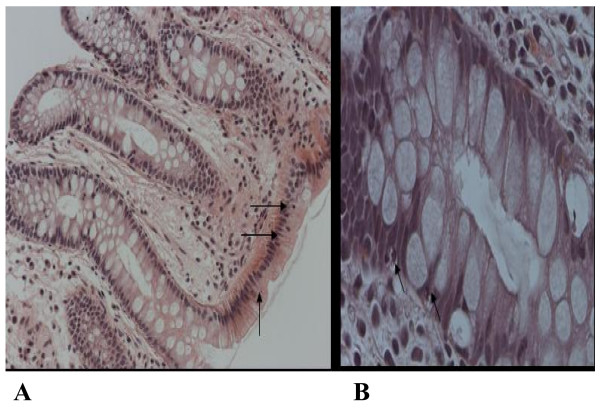
**Apoptosis in H&E stained sections of normal colonic epithelial cells.** (A) 20×, (B) 40×. Arrows indicate apoptotic cells.

Case subjects differed significantly from controls on sex, waist-hip ratio, body mass index (BMI), alcohol, calcium and red meat intake. There was borderline significant difference between cases and controls on age and sex. Compared to controls, adenoma subjects had significantly lower apoptosis and lower tissue IGFBP-3 mRNA (Table 1). Representative gels showing local IGFBP-3 mRNA expression in colonic biopsies are shown in Figure 1. The mean plasma IGFBP-3 did not differ significantly between cases and controls or by gender or race. We divided plasma or tissue IGFBP-3 values into quartiles based on the distribution in controls and evaluated the association between quartiles of plasma or tissue IGFBP-3 and adenomas. The highest quartile was considered as reference. Table 2 shows the unadjusted and adjusted odds ratios (OR) for the associations of plasma IGFBP-3, tissue IGFBP-3 and adenomas. For plasma IGFBP-3 only waist/hip ratio and alcohol remained in the final model. Compared to the highest quartile, the lower three quartiles of plasma IGFBP-3 showed no significant association with adenomas (P_trend _= 0.88). With the highest quartile as reference, low expression of IGFBP-3 mRNA in normal mucosa showed a positive association with risk of adenomas but the results did not reach statistical significance. We observed weak but significant correlation between plasma IGFBP-3 and tissue IGFBP-3 mRNA (Spearman's correlation coefficient 0.12, p = 0.007). The results for the association between apoptosis and adenomas are presented in Table 3. There were no confounders so the final model contained only apoptosis score. Compared to subjects having high apoptosis, those with low apoptosis had a significantly increased risk of adenomas (OR 1.9 95% CI 1.2–2.8). Representative images of apoptosis in H&E stained sections are shown in Figure 2.

**Table 1:** Descriptive Characteristics of Study participants^1^

	Case (n = 185)	Control (n = 484)	p
Age (years, mean, se)	57.3 (0.7)	55.8 (0.5)	0.09
Race – White (%)	82	78	0.28
Sex – Male (%)	59	39	0.0001
Waist/Hip Ratio (mean, se)	0.93 (0.008)	0.89 (0.006)	0.0001
Body Mass Index (BMI, kg/m^2 ^(mean, se))	28.3 (0.4)	27.2 (0.3)	0.04
Smoking Status			
Current (%)	17	10	0.09
Former (%)	37	38	
Never (%)	46	51	
Physical Activity(MET-minutes per day (mean, se))	2627 (63)	2618 (37)	0.89
Family History of CRC^2 ^(% Yes)	17	13	0.30
Alcohol grams per day (mean, se)	12.9 (1.7)	8.2 (0.7)	0.01
Daily Calories (kcal/day (mean, se))	1926 (61)	1866 (34)	0.37
Total Calcium g per day (mean, se)	896 (37)	1039 (25)	0.003
Red Meat oz per day (mean, se)	1.78 (0.11)	1.45 (0.06)	0.01
Fiber g per day (mean, se)	20.2 (0.7)	20.5 (0.4)	0.75
Apoptosis (mean cells/crypt, se)	3.32 (0.10)	3.76 (0.06)	0.0001
Tissue IGFBP-3 (mean, se)	1.13 (0.05)	1.28 (0.05)	0.03
Plasma IGFBP-3 (ng/ml; mean, se)	2012 (68)	2001 (43)	0.89

1. Table includes participants that had tissue IGFBP-3 or plasma IGFBP-3 or both

2. Colorectal cancer (CRC)

**Table 2:** Association between plasma IGFBP-3 or colonic tissue IGFBP-3 and colorectal adenomas

Plasma IGFBP-3	Cases/Controls (n)	Unadjusted^1 ^OR (95% CI)	Adjusted^1,2 ^OR (95% CI)	P
Quartile^4 ^4	44/110	1.0 (Reference)	1.0 (Reference)	
Quartile 3	44/109	1.0 (0.6, 1.7)	0.9 (0.5, 1.7)	
Quartile 2	37/109	0.9 (0.5, 1.5)	0.9 (0.5, 1.7)	
Quartile 1	47/109	1.1 (0.7, 1.8)	1.0 (0.5, 1.9)	
P for linear trend		0.99	0.88	

Tissue IGFBP-3	Cases/Controls (n)	Unadjusted^1 ^OR (95% CI)	Adjusted^1,3 ^OR (95% CI)	

Quartile 4	30/105	1.0 (Reference)	1.0 (Reference)	
Quartile 3	44/105	1.5 (0.9, 2.5)	1.5 (0.8, 2.8)	
Quartile 2	43/103	1.5 (0.9, 2.5)	1.7 (0.9, 3.1)	
Quartile 1	47/103	1.6 (0.9, 2.7)	1.8 (0.9, 3.3)	
P for linear trend		0.07	0.06	

^1 ^Odds ratio (OR) and 95% confidence interval (CI); odds of having colorectal adenomas.

^2 ^Adjusted for waist/hip ratio and alcohol intake for Plasma IGFBP-3.

^3 ^Adjusted for sex and total calcium for Tissue IGFBP-3.

^4 ^Quartile cut points based on distribution of IGFBP-3 levels among control subject. The highest quartile is considered as reference.

**Table 3:** Association between colorectal adenoma and apoptosis.

Apoptosis	Cases/Controls	OR (95% CI)^1^	P
High (Q3 & Q4)^2^	48/163	1.0 (Reference)	--
Low (Q1 & Q2)	89/161	1.9 (1.2, 2.8)	0.003

^1 ^Odds ratio (OR) and 95% confidence intervals (CI); odds of having colorectal adenomas No confounders were identified so final model contained only apoptosis score.

^2 ^Q = quartile; Using the median in controls, apoptosis was divided into low (below the median; quartiles 1 and 2 combined) and high (above the median; quartiles 3 and 4 combined) categories.

We also evaluated the association between plasma IGFBP-3 or tissue IGFBP-3 mRNA expression and apoptosis in the normal colonic mucosa (Table 4). We observed no significant association between plasma IGFBP-3 and apoptosis. Local IGFBP-3 mRNA expression was inversely associated with apoptosis. Compared to the highest quartile, those in the lowest quartile of tissue IGFBP-3 were less likely to have low apoptosis (OR = 0.5, 95% CI 0.3, 0.8).

**Table 4:** Odds ratio and 95% CI for the association between plasma IGFBP-3 or colonic tissue IGFBP-3 and apoptosis

	Apoptosis^1^				
Plasma IGFBP-3	Lower Half/Upper Half (n)	Unadjusted OR (95% CI)^2^	P	Adjusted OR (95% CI)^2,3^	P

Quartile 4	59/44	1.0 (Reference)	--	1.0 (Reference)	--
Quartile 3	57/44	1.0 (0.6, 0.7)	0.90	1.0 (0.6, 0.7)	0.90
Quartile 2	58/45	1.0 (0.6, 0.7)	0.89	1.0 (0.6, 0.7)	0.89
Quartile 1	58/50	0.9 (0.5, 1.5)	0.60	0.9 (0.5, 1.5)	0.60
P for linear trend			0.70		0.70

Colonic tissue IGFBP-3	Lower Half/Upper Half (n)	Unadjusted OR (95% CI)^2^	P	Adjusted OR (95% CI)^2,4^	P

Quartile 4	63/43	1.0 (Reference)	--	1.0 (Reference)	--
Quartile 3	62/51	0.8 (0.5, 1.4)	0.50	0.6 (0.3, 1.1)	0.12
Quartile 2	64/43	1.0 (0.6, 1.8)	0.96	0.8 (0.5, 1.5)	0.57
Quartile 1	51/70	0.5 (0.3, 0.8)	0.01	0.5 (0.3, 0.8)	0.009
P for linear trend			0.10		0.04

^1 ^Number of subjects in each quartile that fall in the lower half or upper of apoptosis

^2 ^Odds ratio (OR) and 95% confidence interval (CI), odds of being in the lower 50^th ^percentile of apoptosis.

^3 ^No potential confounders remained in the final model for Plasma IGFBP-3.

^4 ^Adjusted for red meat for Tissue IGFBP-3.

## Discussion

We assessed whether plasma or tissue IGFBP-3 would predict the risk of colorectal adenomas or low apoptosis in normal colonic mucosa among adenoma cases and non-adenoma controls. Adenomas are intermediate precursors to colorectal cancer therefore, identification of risk factors for adenomas is desirable in order to understand and reduce the risk of colorectal cancer. In this study we found that low apoptosis predicted the risk of colorectal adenomas thus confirming our previous observations [32] in an independent study population. Low expression of tissue IGFBP-3 mRNA correlated with increased risk of adenomas. Surprisingly, the association of tissue IGFBP-3 and apoptosis was not as expected. Low tissue IGFBP-3 was significantly associated with reduced risk of low apoptosis (p = 0.04). There was no association between plasma IGFBP-3 and adenoma risk or apoptosis. Together these data suggest that low levels of tissue IGFBP-3 may increase adenoma risk but is not likely to mediate the association between low apoptosis and increased risk of adenomas. Surprisingly, we did observe significant positive correlation between plasma IGFBP-3 and tissue IGFBP-3 mRNA expression. However, this correlation was weak (r = 0.12) suggesting that colonic tissue IGFBP-3 levels may have only small a contribution to plasma IGFBP-3 given that other tissue sources also influence plasma IGFBP-3 levels. The differential association of tissue IGFBP-3 versus plasma IGFBP-3 with adenoma risk suggests that local IGFBP-3 may better correlate with the functional effects in colonic tissue compared with systemic levels.

Members of the IGF-axis play important roles in regulation of cell growth and apoptosis. Plasma IGF-I is recognized to have a positive association with colorectal cancer. Plasma and tissue IGFBPs modulate the bioavailability of IGF-I and IGF-II and possess anti-proliferative and pro-apoptotic properties. Thus it is essential to understand the role of both plasma and locally expressed IGF-binding proteins in the development of neoplasia. IGF-binding protein-3 (IGFBP-3) is major IGF binding protein and is thought to exert a protective effect through modulation of IGF-I bioactivity as well as through IGF-I independent effects on cell proliferation and apoptosis [36]. The role of local IGFBP-3 in colorectal carcinogenesis is not fully known. Very few human studies, have investigated tissue IGFBP-3 in the human colon and to our knowledge none have assessed the relationship between tissue IGFBP-3 mRNA, adenomas and apoptosis. Our findings that reduced expression of tissue IGFBP-3 mRNA in the normal colon is associated with increased risk of colorectal adenomas are compatible with evidence in experimental animal models indicating that elevated tissue IGFBP-3 protects against colon carcinogenesis [11]. Other studies have shown that elevated IGFBP-3 expression is associated with reduced risk of gastric cancer [3] and prostate cancer [37]. A recent study compared IGFBP-3 mRNA levels and protein localization in grossly normal versus nearby malignant colonic tissue from the same cancer patient [38]. They found that IGFBP-3 mRNA and protein were equivalent in normal epithelium and underlying stroma. In cancer tissue, however, IGFBP-3 mRNA was elevated and this reflected increased expression in the stroma but diminished or absent expression in malignant epithelium [15,38]. However, to our knowledge, no studies have previously evaluated tissue IGFBP-3 mRNA in normal colonic mucosa in relation to adenoma risk. Dietary factors including sodium butyrate [38] and retinoic acid [39] induced IGFBP-3 in gut epithelial cells as well as protect against neoplasia. Multiple mechanisms may control IGFBP-3 actions. For instance, factors such as P53, TGFβ and CDX2 interact with IGFBP-3 to mediate its actions [40-42]. TGFβ induces IGFBP-3 [5,41] and may mediate the effects of IGFBP-3 in the colon [43]. A recent study reported CDX2 as a transcriptional repressor of IGFBP-3, [40] accordingly, it is possible that CDX2 and other factors interact with IGFBP-3 in the colon to promote adenoma development. Our findings indicate that it will be of interest to test if IGFBP-3 levels correlate with dietary factors such as retinoic acid or other local factors such as TGFβ and CDX2.

Observations in experimental animal models and cell lines indicate that IGFBP-3 is pro-apoptotic [6,11,44]. Modulators of apoptosis such as p53, TGFβ, and retinoic acid are associated with increased levels of IGFBP-3 [5,7,13,14]. However, the exact mechanisms underlying the actions of IGFBP-3 are not fully known. Surprisingly, we observed that low expression of IGFBP-3 mRNA in normal colon was associated with high apoptosis. Our findings are intriguing. The interactions of IGFBP-3 with other factors coupled with the complex nature of the IGF-axis may explain our findings. A recent study found that in vitro, IGFBP-3 enhanced TRAIL induced apoptosis in cancer cells but did not have a similar effect on non cancer cells [42]. Therefore, our observations may not be too surprising. Accumulating evidence from other studies suggest that IGFBP-3 alone has weak pro-apoptotic effects but potentiates the pro-apoptotic effects of other factors [38]. In mammary epithelial cells, the pro-apoptotic effects of IGFBP-3 was limited to cancer cells [45]. Our observed association between high tissue IGFBP-3 mRNA and reduced risk of adenomas may therefore reflect different or indirect effects of IGFBP-3 on apoptosis. The precise relationship between tissue IGFBP-3 and apoptosis in the normal human colon appears to be complex. Additional studies are needed to confirm our findings.

Several studies suggest an inverse association between plasma IGFBP-3 and risk of cancer in a number of organs [1,3,20,46,47]. However, this has not been consistently observed by all studies [29]. In some studies, elevated plasma IGF-I was associated with increased risk of colorectal adenomas while inverse associations were reported for plasma IGFBP-3 [25,48]. Teramukai et al. 2002 [49] also found a modest inverse association between colorectal adenomas and high plasma levels of IGFBP-3. We observed no significant association between plasma IGFBP-3 and adenomas or plasma IGFBP-3 and apoptosis in this patient population. Our study highlights the importance of considering plasma as well as tissue IGFBP-3 in the early stages of colon tumor development, consistent with recent reports on tissue IGFBP-3 in colon cancer [38].

## Conclusion

In summary, we found that tissue IGFBP-3 but not plasma IGFBP-3 was associated with modestly elevated risk of colorectal adenomas. Our findings suggest that low local IGFBP-3 expression may increase adenoma risk but is not likely to mediate the relationship between adenomas and apoptosis.

## Abbreviations

IGFBP-3: Insulin-like growth factor Binding protein-3; IGF-I: Insulin-like growth factor; IGFBPs: Insulin-like binding proteins; CRC: colorectal cancer; ALS: Acid labile subunit.

## Competing interests

The authors declare that they have no competing interests.

## Authors' contributions

TOK contributed to study conception, design, and drafted the manuscript; RSS and PKL were responsible for study conception, design, and revision of manuscript draft for intellectual content; JGS assisted with study coordination, RT-PCR assay optimization, data interpretation and manuscript preparation; JTW performed pathological assessment of colonic specimens to determine case-control status of patients and contributed to manuscript preparation; MP assisted with apoptosis evaluation, RT-PCR and manuscript preparation, OO and MMcD performed RNA extractions, and contributed to data acquisition and manuscript preparation; JG assisted with data management, statistical analysis and drafting the manuscript. All authors read and approved the final manuscript

## Pre-publication history

The pre-publication history for this paper can be accessed here:


